# Mechanisms Underlying the Action of Ziziphi Spinosae Semen in the Treatment of Insomnia: A Study Involving Network Pharmacology and Experimental Validation

**DOI:** 10.3389/fphar.2021.752211

**Published:** 2021-12-24

**Authors:** Zhenhua Bian, Wenming Zhang, Jingyue Tang, Qianqian Fei, Minmin Hu, Xiaowei Chen, Lianlin Su, Chenghao Fei, De Ji, Chunqin Mao, Huangjin Tong, Xiaohang Yuan, Tulin Lu

**Affiliations:** ^1^ College of Pharmacy, Nanjing University of Chinese Medicine, Nanjing, China; ^2^ Department of Pharmacy, Wuxi TCM Hospital Affiliated to Nanjing University of Chinese Medicine, Wuxi, China

**Keywords:** Ziziphi Spinosae Semen, insomnia, network pharmacology, targets, pharmacological mechanisms

## Abstract

**Purpose:** This study aimed to investigate the potential mechanisms and related bioactive components of ZSS for the treatment of insomnia.

**Method:** The insomnia model of rat induced by PCPA was established. After oral administration of ZSS extract, the general morphological observation, pentobarbital sodium-induced sleep test and histopathological evaluation were carried out. Network pharmacology, assisted by UHPLC-Q-Exactive-MS/MS analysis, was developed to identify the targets of ZSS in the treatment of insomnia, as well as the corresponding signaling pathways. In addition, we validated the identified targets and pathways by RT-qPCR and immunohistochemical analysis.

**Results:** The pentobarbital sodium-induced sleep test, determination of 5-HT and GABA levles in hypothalamic tissues and HE staining showed that ZSS extract was an effective treatment for insomnia. Network pharmacology analysis identified a total of 19 candidate bioactive ingredients in ZSS extract, along with 433 potentially related targets. Next, we performed protein-protein interaction (PPI), MCODE clustering analysis, GO functional enrichment analysis, KEGG pathway enrichment analysis, and ingredient-target-pathway (I-T-P) sub-networks analysis. These methods allowed us to investigate the synergistic therapeutic effects of crucial pathways, including the serotonergic and GABAergic synapse pathways. Our analyses revealed that palmitic acid, coclaurine, jujuboside A, N-nornuciferine, caaverine, magnoflorine, jujuboside B, and betulinic acid, all played key roles in the regulation of these crucial pathways. Finally, we used the PCPA-induced insomnia in rats to validate the data generated by network pharmacology; these *in vivo* experiments clearly showed that pathways associated with the serotonergic and GABAergic system were activated in the rats model. Furthermore, ZSS treatment significantly suppressed high levels of HTR1A, GABRA1, and GABRG2 expression in the hypothalamus and reduced the expression levels of HTR2A.

**Conclusion:** Based on the combination of comprehensive network pharmacology and *in vivo* experiments, we successfully identified the potential pharmacological mechanisms underlying the action of ZSS in the treatment of insomnia. The results provide a theoretical basis for further development and utilization of ZSS, and also provide support for the development of innovative drugs for the treatment of insomnia.

## Introduction

Insomnia is a common sleep disorder that is characterized by sustained difficulty initiating or maintaining sleep. The global prevalence of insomnia symptoms range from 8 to 40% (Vgontzas and Fernandez-Mendoza, 2013). Research has shown that insomnia frequently causes other psychological and physical disorders, including depression, anxiety, hypertension, diabetes, cardiovascular diseases, and cerebrovascular diseases (Morin et al., 2014; Horsch et al., 2017). Numerous sedative-hypnotic drugs have been used in the clinic, including benzodiazepines, antihistamines, and antidepressants; however, these drugs have common side effects, such as dizziness, lethargy, and physical dependence (Zhou et al., 2018). In addition, Chinese herbal medicine has been historically used to treat insomnia and is now a recognized therapeutic used across the world (Shi et al., 2014).

Ziziphi Spinosae Semen (ZSS), known as suan zao ren in China, has been widely used to manage insomnia and palpitations in Traditional Chinese Medicine (Yeung et al., 2012; Rodríguez Villanueva and Rodríguez Villanueva, 2017; Shergis et al., 2017; Zhou et al., 2018). Modern pharmacological research indicates that ZSS exhibits good sedative and hypnotic effects on the central nervous system (Xiao et al., 2018). There are many prescriptions containing ZSS as a raw medical material in the Chinese Pharmacopoeia; these are commonly used to treat palpitations and insomnia. Over 150 different components have been separated and identified from ZSS, including saponins, flavonoids, alkaloids, and polysaccharides. Total saponins and compounds from ZSS are well known for their significant sedative and hypnotic effects (Du et al., 2020). Until now, the pharmacological investigation of total saponins for the treatment of insomnia has mainly focused on jujubosides, jujuboside A, jujuboside B, and other monomers. A previous research study showed that jujubosides, the main saponins of ZSS, significantly reduced the spontaneous activity of mice by regulating the serotonin system (He et al., 2020). It has also been reported that jujuboside A can inhibit the formation of the hippocampus *via* a glutamate-mediated excitatory signaling pathway (Zhang et al., 2003). Jujubosides can also modulate the expression of γ-amino-butyric acid A (GABA_A_) receptor subunits in hippocampal neurons (You et al., 2010). Other studies have shown that jujuboside B can up-regulate the expression of GABA_A_ receptors and increase the frequency of chloride channel opening, thus creating a hypnotic effect (Song et al., 2017). It has been shown that the total flavonoids of ZSS can extensively reduce the spontaneous activity of mice and prolong their sleep time (Jiang et al., 2007). Spinosin, the main flavonoid of ZSS, can enhance pentobarbital-induced sleep by regulating the serotonergic system (Wang et al., 2008). However, it is important that we identify potential mechanisms for the overall effects of ZSS if we are to create advanced approached for treating insomnia.

**FIGURE 1:**
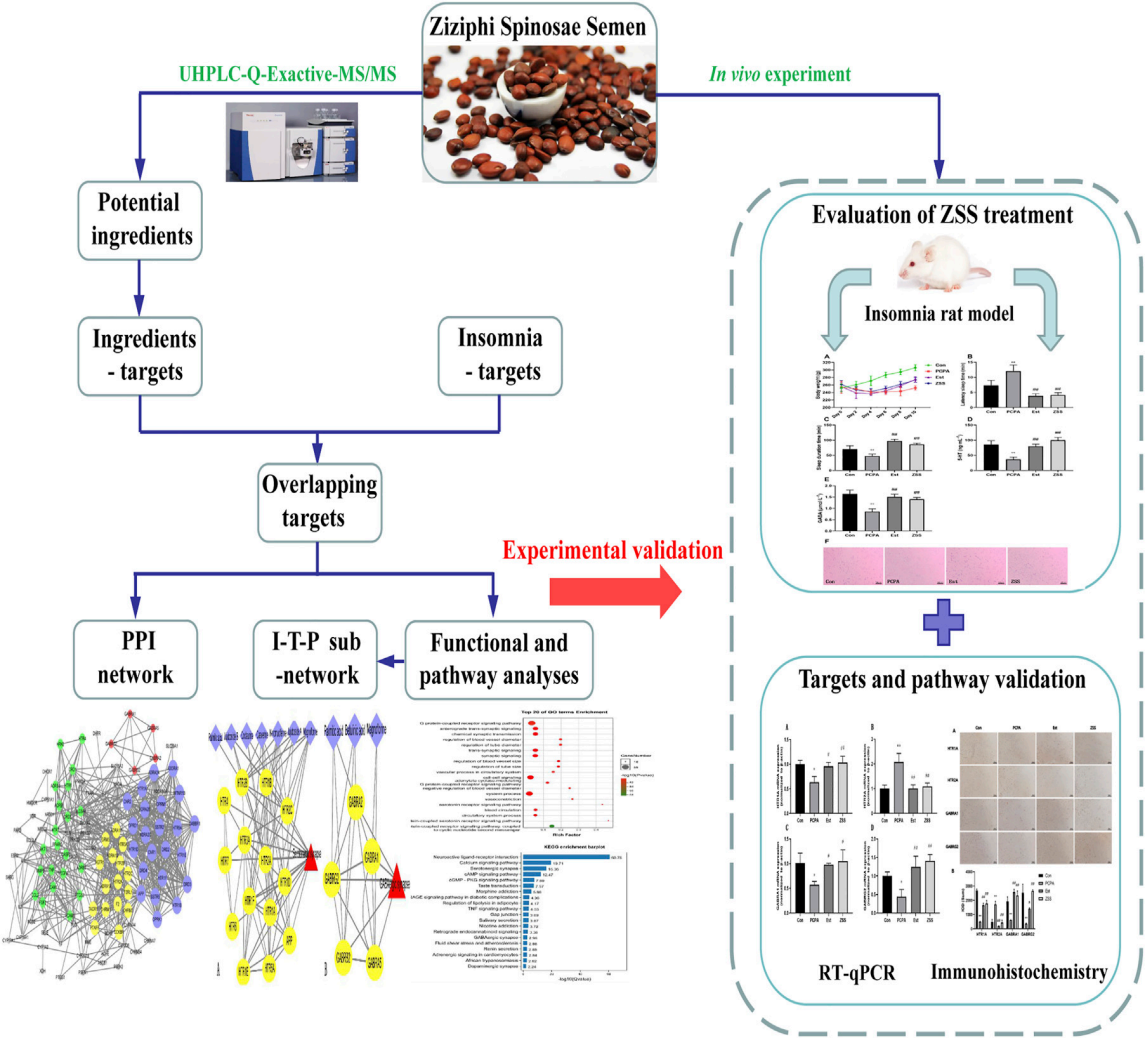
The workflow plan used to investigate the effects of ZSS on insomnia using network pharmacology and an experimental validation approach.

In this study, we used network pharmacology assisted by UHPLC-Q-Exactive-MS/MS analysis to predict the active ingredients and candidate targets fo ZSS. We also used network construction to investigate the active mechanisms underlying the effect of ZSS treatment on insomnia. Finally, we validated the proposed active mechanisms of ZSS in a PCPA-induced insomnia rat model by RT-qRCR and immunohistochemical analysis. The research procedure is shown in Figure 1.

## Materials and Methods

### Chemicals and Reagents

Ziziphi Spinosae Semen (ZSS) was purchased from Jiangsu Yabang Chinese Herbal Medicine Co., Ltd. (Changzhou, China). Jujuboside A (batch number P13J9S65562), Jujuboside B (batch number C28A10S87087), Spinosin (batch number P09D11F133853), 6ʹʹʹ-Feruloylspinosin (batch number P04J12S136476), Betulinic acid (batch number R17F11F108704) were obtained from Shanghai yuanye Biological Technology Co., Ltd. (Shanghai, China). Pentobarbital sodium was purchased from Tianjin Yifang Technology Co., Ltd. (Tianjin, China). *p*-chlorophenylalanine (PCPA) was supplied by Sinopharm Chemical Reagent Co., Ltd. (Shanghai, China). Acetonitrile, Formic acid and methanol (LC-MS grade) were obtained from Thermo Fisher Scientific (New Jersey, United States). Ammonium formate (LC-MS grade) was purchased from Sigma Aldrich (St. Louis, United States) while 2-chlorobenzalanine was purchased from Shanghai Aladdin Biochemical Technology Co., Ltd. (Shanghai, China). Ultrapure water was purified with a Milli-Q purification system (Milford, MA, United States). Other chemicals and reagents were all analytical grade.

### Plant Extract Preparation

ZSS was formally identified as *Ziziphus jujuba* Mill. var. *spinosa* (Bunge) Hu ex H. F. Chou by Professor Tu-Lin Lu (Nanjing University of Chinese Medicine). The ZSS voucher specimen (ZSS-19121601) was deposited in the Traditional Chinese Medicine laboratory, Wuxi Traditional Chinese Medicine Hospital Affiliated to Nanjing University of Chinese Medicine under closed and dry conditions at 25 ± 5°C. A dried sample of ZSS was crushed and extracted with petroleum ether (60–90°C) for 2 h and the extraction repeated two further times. After degreasing, we added 8 volumes of 70% alcohol to the ZSS; the mixture was then fluxed three times (1.5 h each time) and the final filtrates were combined. The filtered extract was concentrated under a vacuum and then dried by a rotary evaporator (Shang et al., 2020). The total yield of alcohol extract from ZSS was 14.94% (w/w).

### UHPLC-Q-Exactive-MS/MS Analysis of ZSS Extract

#### Sample Preparation

First, we weighed 6.9 mg of ZSS extract and mixed this with 5 ml of methanol. This was then ultrasonicated at room temperature for 15 min (300 W, 40 KHZ) and centrifuged for 10 min at 12,000 rpm. Finally, the supernatants were filtered through a 0.22 µm membrane to obtain prepared samples for UHPLC-Q-Exactive-MS analysis.

#### UHPLC-Q-Exactive-MS/MS Conditions

Chromatographic separation was accomplished in a Vanquis UHPLC system (Thermo Fischer Scientific, Waltham, MA, United States) equipped with a Thermo Fischer Scientific Hypersll GOLD (100 × 2.1 mm, 1.8 µm) column maintained at 35°C with a flow rate of 0.3 ml/min. The temperature of the autosampler was 5°C. Gradient elution of analytes was carried out with 0.1% acetic acid in 10 mM ammonium acetate buffer solution (A) and acetonitrile (B). We injected 1 µl of each sample after equilibration. An increasing linear gradient of solvent B (v/v) was then applied, as follows: 0–10 min, 5–20% B; 10–14 min, 20–25% B; 14–25 min, 25–35% B; 25–30 min, 35–100% B; 30–31 min, 100–100% B; 31–32 min, 100–5% B; 32–35 min, 5–5% B.

The ESI-MS^n^ experiments were executed on a Thermo Q Exactive mass spectrometer (Thermo Fischer Scientific, Massachusetts, United States) with spray voltages of 3.5 and −3.0 kV in positive and negative modes, respectively. Sheath gas and auxiliary gas were set at 40 and 15 arbitrary units, respectively. The capillary temperature was 320°C. The analyzer scanned over a mass range of m/z 150–2,000 Da for a full scan at a mass resolution of 70,000. Data dependent acquisition (DDA) MS/MS experiments were performed with HCD scans. Dynamic exclusion was implemented to remove some unnecessary information in MS/MS spectra.

#### Data Processing

The analysis of UHPLC-MS data was performed using Thermo Xcalibur Version 4.1 software (Thermo Fischer Scientific, Massachusetts, United States). We tentatively identified the compounds of ZSS by considering a range of factors, including molecular weight, retention time, fragment information obtained from the MS/MS model, further matching annotation in our in-house database of compounds, along with previous literature and standard references.

#### Animal Experimentation

Male Sprague-Dawley rats (200 ± 20 g) were obtained from SPF (Beijing) Biotechnology Co., Ltd (Permission No. SCXK (jing) 2019–0010). All animals were housed in a breeding environment (12 h light-dark cycle, 25 ± 2°C, and 55 ± 5% relative humidity). All rats had free access to water and food. This animal research was approved by the Ethics Committee of Wuxi Hospital of Traditional Chinese Medicine (Approval ID: SKJJ2020011707) and conformed to animal welfare regulations and the ethical principles for animal protection and the relevant provisions put forward by the National Experimental Animal Welfare Ethics guidelines.

After 7 days of acclimation, the rats were randomly divided into four groups (6 rats per group): a normal group, a model group, an estazolam group, and a ZSS group. The PCPA-induced model of insomnia was established using a method that was described previously (Si et al., 2020). Rats in the model group, the estazolam group, and the ZSS group, were intraperitoneally injected with PCPA (350 mg/kg) once a day for 3 days. The normal group received the same amount of physiological saline. On the fourth day, the ZSS group was orally administered with ZSS alcohol extract (dissolved in normal saline) at a dose of 403.38 mg/kg (equivalent to a crude drug dose of 2.7 g/kg) once a day for 7 days (Du et al., 2019; Hua et al., 2021). The human equivalent dose (HED) of the dose is 0.43 g/kg, which is 2 times of the clinical dosage (Food and Drug Administration, 2005). The estazolam group was administered with 0.5 mg/kg of estazolam (dissolved in normal saline) once a day for 7 days. Rats in the other two groups were treated with an equal volume of physiological saline. At the end of the animal experiment and after fasting for 12 h, all rats were anaesthetized by an intraperitoneal injection of pentobarbital sodium (45 mg/kg). Blood samples were then collected from the abdominal aorta and hypothalamic tissues were quickly removed.

#### Pentobarbital Sodium-Induced Sleep Test

This experiment was carried out 30 min after the last drug administration. Rats were placed on their backs following an intraperitoneal injection of pentobarbital sodium (35 mg/kg), and then monitored the rats for signs of sleeping. Our main criterion for sleep was that the rats lost their righting reflex for more than 1 min. Sleep latency was recorded as the time between pentobarbital sodium injection and the loss of the righting reflex. Sleep duration was recorded from the loss of the righting reflex until recovery (Xu et al., 2019).

#### Assay for Hypothalamic 5-HT and GABA

The hypothalamic samples were homogenized with ice-cold PBS (w/v, 1:9). The homogenate was centrifuged at 3,000 rpm for 10 min at 4°C. The supernatant was collected. The levels of 5-HT and GABA in hypothalamic tissues were measured using ELISA kits according to the manufacturer’s instructions (Nanjing JinTing Biotechnology Co., Ltd. Nanjing, China).

#### Histopathological Examination

Hypothalamic tissues were fixed in 4% paraformaldehyde, dehydrated, embedded in paraffin, and then sectioned. Sections (4 μm thick) were then dewaxed to water, and stained with hematoxylin and eosin (HE). Finally, hypothalamic lesions were observed by microscopy, as described previously (Shen et al., 2020).

### Network Pharmacology

#### The Screening of Candidate Ingredients

The phytochemicals identified by UHPLC-Q-Exactive-MS/MS were used for network pharmacology investigations. We used Lipinski’s rule of five related parameters to screen the active compounds: a molecular weight (MW) ≤ 500, an octanol-water partition coefficient log P (ALogP) ≤ 5, a hydrogen bond donor count (Hdon) ≤ 5, and a hydrogen bond acceptor count (Hacc) ≤ 10. The principle of screening candidate ingredients was to meet at least two of the parameters (Wang et al., 2020). Some compounds (e.g., jujuboside A) exhibited low values with explicit pharmacological effects; these were also selected for further study.

#### Analysis of Putative Targets

We used BATMAN-TCM (http://bionet.ncpsb.org.cn/batman-tcm/) (Ji et al., 2019) and the SwissTargetPrediction database (http://www.swisstargetprediction.ch/) (Gfeller et al., 2014) to identify the relevant biological targets of candidate ingredients from ZSS. We also retrieved disease-associated targets from the GeneCards database (https://www.genecards.org/) by using “insomnia” and “sedation and hypnosis” as keywords (Hu et al., 2020). Then, the overlapping targets between ingredient- and insomnia-associated targets were then visualized by creating Venn diagrams (https://bioinfogp.cnb.csic.es/tools/venny/index.html) (Huang et al., 2020). In addition, these overlapping targets were introduced into the STRING database (https://www.string-db.org/) to investigate protein-protein interaction (PPI) relationships; this allowed us to identify targets that were closely related to insomnia (Liang et al., 2021); the protein interaction selection score was set to >0.6. Cytoscape version 3.6.1 software (Free Software Foundation, Inc., Boston, MA, United States) was used to visualize the PPI network. Then, we used the MCODE plugin to conduct cluster analysis of the targets showing high levels of interaction (Zhang et al., 2019).

#### GO and KEGG Pathway Enrichment Analyses

Next, the core targets obtained from MCODE cluster analysis were imported into Omicshare tools (https://www.omicshare.com/). We then performed Gene Ontology (GO) and Kyoto Encyclopedia of Genes and Genomes (KEGG) pathway enrichment analyses for the core targets and identified the key biological functions (biological processes, molecular functions, and cellular components) and related signaling pathways (Wu et al., 2020). Finally, an ingredient-target-pathway (I-T-P) network was generated by Cytoscape version 3.6.1 software, which featured a number of relationships, including the active chemical ingredients of ZSS, the core targets, and the enriched signaling pathways (An et al., 2020).

### Experimental Validation

#### RT-qPCR Experiment

Total RNA was extracted from the rat hypothalamus with Trizol reagent (Invitrogen, Carlsbad, CA, United States). cDNA was then synthetized with a reverse transcription kit (TaKaRa, Dalian, China) by GeneExplorer PCR (Bioer Technology, Hangzhou, China). Next, the cDNA was used as a target for amplification using the TB Green Premix PCR Kit (TaKaRa, Dalian, China) and a LightCycler 480 Ⅱ (Roche, Rotkreuz, Switzerland). The real-time PCR thermal cycling protocol was as follows: 95°C for 5 min, followed by 50 cycles of 95°C for 10 s, 60°C for 10 s and 72°C for 10 s. The primer sequences were synthesized by Sangon Biotech Co., Ltd. (Shanghai, China), as follows: β-actin (Forward) 5′-CCT​CAC​TGT​CCA​CCT​TCC​A-3′ and (Reverse) 5′-GGG​TGT​AAA​ACG​CAG​CTC​A-3´; HTR1A (Forward) 5′-GGG​CAA​CTC​CAA​AGA​GCA-3′ and (Reverse) 5′-TCA​CCG​TCT​TCC​TTT​CAC​G-3´; HTR2A (Forward) 5′-TTC​CTT​GTC​ATG​CCT​GTG​T-3′ and (Reverse) 5′-ATA​GCG​GTC​CAG​GGA​GAT-3´; GABRA1 (Forward) 5′-GAC​TAT​CTT​TGG​GCC​TGG​A-3′ and (Reverse) 5′-CAT​CTT​GGG​AGG​GCT​GT-3´; GABRG2 (Forward) 5′-ACA​ATG​CCA​CCC​ACC​TT-3′ and (Reverse) 5′-TAT​CCT​CCC​GTG​TCT​CCA-3´. The relative expression of the target genes were normalized to the threshold cycle (CT) value of β-actin, and the data analysis was performed using the 2^-△△Ct^ method (Poh et al., 2017). Real-time PCR was performed for each sample in three replicates.

#### Immunohistochemistry

Paraffin-embedded rat hypothalamic tissues were sectioned and dewaxed to water. Then, high pressure boiling was used for antigen retrieval and endogenous catalase was blocked by 3% H_2_O_2_. Next, sections (4 μm thick) were incubated overnight at 4°C with HTR1A (PA5-75267, 1:200) (Invitrogen, Carlsbad, CA, United States), HTR2A (PA5-95288, 1:200) (Invitrogen, Carlsbad, CA, United States), GABRA1 (ab94585, 1:200) (Abcam, United Kingdom) and GABRG2 (ab87328, 1:200) (Abcam, United Kingdom) antibodies. The following morning, sections were washed and then incubated for 20 min at 37°C with HRP-conjugated secondary antibodies (goat anti-rabbit IgG H&L, ab205718, Abcam, 1:2,000), followed by DAB and hematoxylin staining. Finally, the stained sections were imaged and observed by NIKON ECLIPSE CI microscopy (Nikon, Japan). The integrated optical density (IOD) of the expression levels of target proteins/positive cells were then quantified with Image Pro Plus version 7.0 software (Media Cybernetics, Inc., MD, United States) (Jin et al., 2020).

#### Statistics

Experimental data are shown as mean ± standard deviation (SD). One-way analysis of variance (ANOVA) was used for all statistical analysis followed by a Dunnettʹs test and performed in GraphPad Prism version 8 (GraphPad Software Inc., San Diego, CA, United States). *p*-values < 0.05 was considered to be statistically significant.

## Results

### UHPLC-Q-Exactive-MS Analysis of ZSS Extract

UHPLC-Q-Exactive-MS identified a total of 34 phytochemicals in ZSS extract, including twenty-four flavonoids, two triterpenoid saponins, two triterpene acids, four alkaloids, and two fatty acids (Table 1). Five of these compounds (spinosin, 6ʹʹʹ-feruloylspinosin, jujuboside A, jujuboside B, and betulinic acid) were identified by comparison to standard references; the others were identified by comparison with literature.

**TABLE 1:** Identified ingredients in ZSS extract.

NO.	RT (min)	Compound name	Formula	MS	Error (ppm)	MS/MS
1	7.07	Magnoflorine	C_20_H_24_NO_4_	342.1697 [M]^+^	−0.877	297.1119,282.0885,265.0858,58.0660
2	7.29	Coclaurine	C_17_H_19_NO_3_	286.1432 [M + H]^+^	−2.097	269.1170,237.0908,219.0807,175.0753,107.0495
3	7.50	Vicenin Ⅱ	C_27_H_30_O_15_	593.1527 [M-H]^-^	2.697	503.1214,473.1101,383.0780,353.0673
4	9.31	6‴-(4‴'-O-glc)-vanilloylspinosin	C_42_H_48_O_23_	921.2646 [M + H]^+^	−0.217	351.0860,327.0861,297.0757,151.0390
5	9.45	Isovitexin-2ʹʹ-O-β-D-glucopy-ranoside	C_27_H_30_O_15_	593.1536 [M-H]^-^	4.215	413.0886,293.0460
6	9.93	Zivulgarin	C_28_H_32_O_15_	609.1801 [M + H]^+^	−2.134	447.1288,351.0871,327.0861,297.0755,285.0757
7	10.05	Camelliaside B	C_32_H_38_O_19_	725.194 [M-H]^-^	0.827	575.1411,284.0331,255.0297
8^a^	10.09	Spinosin	C_28_H_32_O_15_	609.1799 [M + H]^+^	−2.462	447.1291,429.1185,411.1071,393.0966,381.0961, 351.0862,327.0862,297.0757,285.0757
9	10.11	Isospinosin	C_28_H_32_O_15_	607.1682 [M-H]^-^	2.306	487.1257,445.1151,427.1041,324.0644,307.0617,292.0380
10	10.14	Isovitexin	C_21_H_20_O_10_	433.1115 [M + H]^+^	−3.463	397.0923,379.0818,337.0707,313.0707,283.0600
11	10.69	Swertisin	C_22_H_22_O_10_	445.1144 [M-H]^-^	0.899	325.0724,297.0410,282.0539
12	11.11	Caaverine	C_17_H_17_NO_2_	268.1327 [M + H]^+^	−1.865	251.1065,219.0804,191.0854
13	11.14	6ʹʹʹ-Pyridyloylspinosin	C_34_H_35_NO_16_	714.2020 [M + H]^+^	−1.260	351.0865, 327.0862, 323.0930,297.0759,124.0395
14	11.46	Kaempferol-3-rutinoside	C_27_H_30_O_15_	593.1524 [M-H]^-^	2.192	285.0407
15	11.73	6ʹʹʹ-*p*-Hydroxylbenzoylspinosin	C_35_H_36_O_17_	727.1891 [M-H]^-^	1.650	427.1042,325.0724,307.0616,239.0563,179.0345,137.0236
16	11.79	Isovitexin-2″-O-(6-feruloyl) -glucopyranoside	C_37_H_38_O_18_	769.1995 [M-H]^-^	1.300	413.0883,341.0675,311.0566,293.0462,235.0613,193.0500
17	12.00	6‴-O-(3S-1-N-β-d-glucopyranosyl -2-oxo-3-hydroxy-indole-3-acetyl) spinosin	C_44_H_49_NO_23_	958.2634 [M-H]^-^	1.252	649.1790,607.1710,487.1236,469.1152,307.0618,146.0238
18	12.31	6‴-O-(3R-1-N-β-d-glucopyranosyl -2-oxo-3-hydroxy-indole-3-acetyl) spinosin	C_44_H_49_NO_23_	958.2631 [M-H]^-^	0.939	649.1757,607.1664,427.1036,307.0636
19	12.34	6ʹʹʹ-Sinapoylspinosin	C_39_H_42_O_19_	813.2258 [M-H]^-^	1.230	427.1042,307.0616
20	12.70	6ʹʹʹ-*p*-Coumaloylspinosin	C_37_H_38_O_17_	753.2056 [M-H]^-^	2.655	633.1624,607.1690,445.1169,427.1041,325.0724, 307.0610,265.0721,205.0504
21^a^	12.72	6ʹʹʹ-Feruloylspinosin	C_38_H_40_O_18_	785.2261 [M + H]^+^	−3.311	505.7764,411.1066,393.0963,351.0869,327.0859, 321.0950,297.0747,177.0547
22	13.44	6‴-(N-β-d-glucopyranosyl)-2‴',3‴' -dihydro-2‴'-oxo-3‴'-yl-acetate spinosin	C_44_H_49_NO_22_	944.2802 [M + H]^+^	6.460	393.0969,351.0863,327.0862,297.0765
23	13.67	Isomer of NO.22	C_44_H_49_NO_22_	944.2805 [M + H]^+^	6.778	393.0960,351.0872,327.0860,297.0756
24	14.30	6-(−) -phaseolspinosin	C_43_H_50_O_19_	869.2889 [M-H]^-^	1.841	779.7828,607.1709,545.9795,510.7946,477.1872,427.1047
25	14.71	6ʹʹʹ-benzoylspinosin	C_35_H_36_O_16_	713.2072 [M + H]^+^	−0.561	327.0860,351.0859,297.0739
26	15.46	N-nornuciferine	C_18_H_19_NO_2_	282.1481 [M + H]^+^	−2.835	265.1221,250.0986,234.1037
27	15.48	6ʹʹ-O-(3-glc-indole-acetyl)- 6ʹʹʹ-feruloylspinosin	C_54_H_57_NO_25_	1120.3273 [M + H]^+^	−1.696	393.0963,351.0861,327.0860,297.0745,285.0760, 177.0546,146.0601,145.0285
28	15.65	Isomer of NO.27	C_54_H_57_NO_25_	1120.3273 [M + H]^+^	−1.696	393.0963,351.0861,327.0860,297.0745,285.0760, 177.0546,146.0601,145.0285
29^a^	23.98	Jujuboside A	C_58_H_94_O_26_	1205.5967 [M-H]^-^	0.498	1073.5563,911.5052,749.4493,603.3905
30^a^	26.58	Jujuboside B	C_52_H_84_O_21_	1043.5446 [M-H]^-^	1.342	911.5043,749.4492, 603.3910
31	29.38	Ceanothic acid	C_30_H_46_O_5_	485.3280 [M-H]^-^	1.648	423.3280
32^a^	31.19	Betulinic acid	C_30_H_48_O_3_	455.3538 [M-H]^-^	1.757	
33	32.29	Palmitic acid	C_16_H_32_O_2_	255.2332 [M-H]^-^	1.175	
34	32.35	Oleic acid	C_18_H_34_O_2_	281.2491 [M-H]^-^	1.778	

a -identified by standard references.

### General Status

**FIGURE 2:**
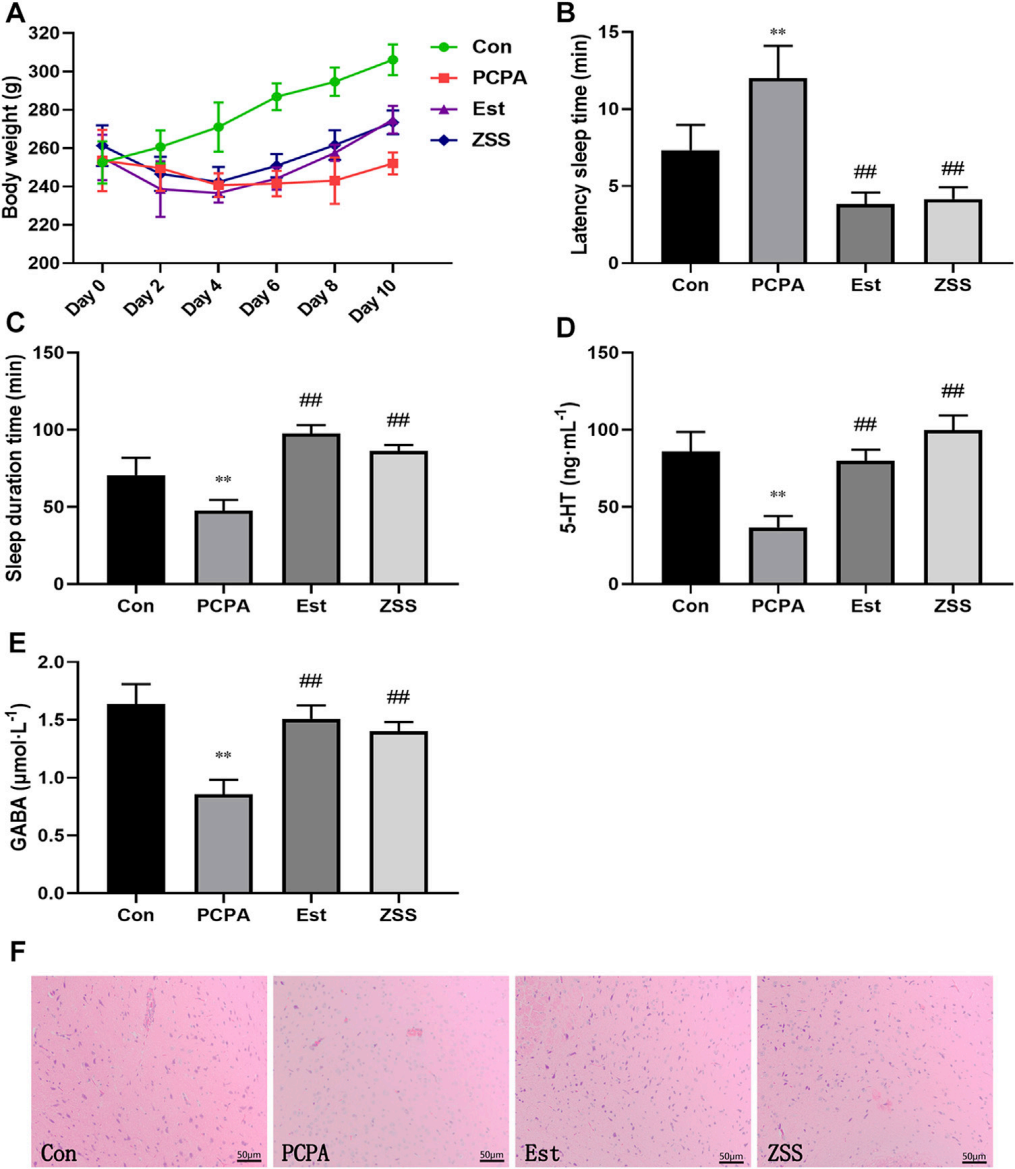
The effects of ZSS extract on body weight **(A)**, pentobarbital sodium-induced sleep latency **(B)** and sleep duration **(C)**, the levels of 5-HT **(D)** and GABA **(E)**. Histopathological observation of the hypothalamus at ×200 magnification **(F)**. Con, control group; PCPA, PCPA-induced insomnia model group; Est, estazolam-positive group; ZSS, alcohol extract of Ziziphi Spinosae Semen group. ^**^ *p* < 0.01 compared with the control group, ^##^ *p* < 0.01 compared with the model group. Data shows mean ± SD values and n = 6.

Twelve to 48 h after the injection of PCPA, most of the rats began to show hyperactivity; they also showed increased levels of sensitivity and aggressiveness. Their fur became dry and dull and the color of their toenails began to turn white or yellow. Over time, the rats in the model group began to show fatigue, characteristic dullness developed in their fur, and they showed a slow response to external stimulation. Eventually, the rats showed serious fatigue and had begun to gain weight gradually. We found that the fur color and response to external stimulation were improved in the ZSS group and the estazolam group. Rats in the normal control group showed a normal state throughout the entire experiment. There was a significant difference in weight gain when compared between the control group and the PCPA group (*p* < 0.05) (Figure 2A). The weight of rats in the ZSS and estazolam treatment groups were significantly higher than that in the PCPA group (*p* < 0.05).

### Effects of ZSS on Pentobarbital Sodium-Induced Sleep

The latency sleep time of rats in the estazolam group and the ZSS group was significantly lower (*p* < 0.01) than that in the PCPA group (Figure 2B). Treatment with estazolam or ZSS extract significantly prolonged the total sleep time (*p* < 0.01) when compared with the PCPA group (Figure 2C).

### Effects of ZSS on Hypothalamic 5-HT and GABA in PCPA-Induced Insomnia Rat

As shown in Figures 2D,E, the levels of 5-HT and GABA in the hypothalamus of the PCPA group were significantly decreased as compared to the control group (*p* < 0.01). Compared with the PCPA group, oral administration of SCE remarkably increased the levels of 5-HT and GABA (*p* < 0.01).

### Histopathological Observation

In the control group, there was an abundance of hypothalamic nerve cells; these had a clear shape and were evenly distributed. In the PCPA group, cells were deformed, loosely arranged; some had even disappeared. These pathological changes were restored when rats were treated with ZSS extract and estazolam (Figure 2F).

### Network Pharmacology

#### Candidate Ingredients and Potential Targets of ZSS

**FIGURE 3:**
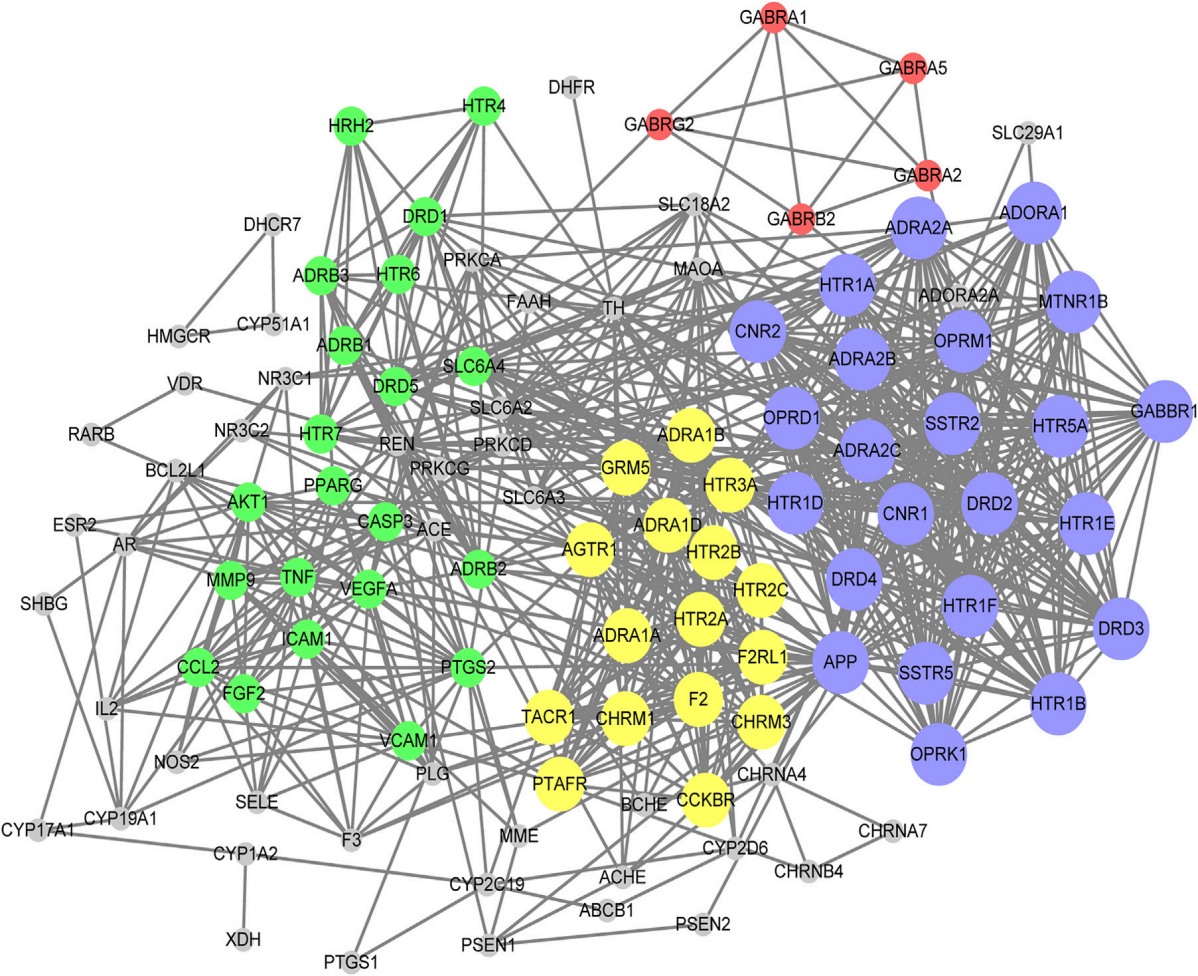
PPI network of the potential targets for ZSS in the treatment of insomnia. Different clusters are represented by different colors. For each cluster, node size is directly proportional to the MCODE score.

According to Lipinski’s rule, our network pharmacology study identified 21 candidate compounds (Table 2). Using associated databases, we failed to identify related targets for 6ʹʹʹ-Sinapoylspinosin and 6ʹʹʹ-*p*-Coumaloylspinosin. Ultimately, we identified 19 active compounds and 433 compound-related targets (Supplementary Table S1). In total, 504 insomnia-associated targets were acquired from the GeneCards database (Supplementary Table S2). After overlapping the ZSS-associated targets and the insomnia-associated targets, we identified 118 targets potential targets for ZSS in the treatment of insomnia (Supplementary Table S3). Next, we created a PPI network and performed MCODE clustering analysis in Cytoscape to identify the way the potential targets interacted and to identify kernel targets. We identified 111 nodes and 787 edges in the PPI network (Figure 3). The clustering coefficient and average neighborhood number were 0.611 and 14.180, respectively. MCODE clustering analysis identified 4 clusters. As shown in Table 3, 65 key targets were identified from 4 clusters, thus representing potential core targets for ZSS in the treatment of insomnia. Most of these targets were neuroactive ligand receptors, including serotonin receptors (e.g., HTR1A, HTR1B, HTR1E, HTR1D, HTR1F, HTR2A, HTR2B, HTR2C, HTR3A and HTR5A), GABA_A_ receptors (e.g., GABRA1, GABRA2, GABRA5, GABRB2 and GABRG2), dopamine receptors (e.g., DRD1, DRD2, DRD3, DRD4 and DRD5), adrenergic receptor (e.g., ADRA1A, ADRA1B, ADRA1D, ADRA2A, ADRA2B and ADRA2C), cannabinoid signaling (e.g. CNR1 and CNR2), and muscarinic acetylcholine receptors (e.g. CHRM1 and CHRM3).

**TABLE 2:** The parameters of drug-likeness of candidate compounds.

NO.	Compound	Molecular weight	ALogP	Hydrogen bond donor count	Hydrogen bond acceptor count
1	Magnoflorine	342.4	2.7	2	4
2	Coclaurine	285.34	2.6	3	4
3	Vicenin Ⅱ	594.5	−2.3	11	15
4	Zivulgarin	608.5	−1.6	9	15
5	Camelliaside B	726.6	−2.5	11	19
6	Spinosin	608.5	−1.1	9	15
7	Isospinosin	608.5	−1.1	9	15
8	Isovitexin	432.4	0.2	7	10
9	Swertisin	446.4	0.5	6	10
10	Caaverine	267.32	2.6	2	3
11	Kaempferol-3-rutinoside	594.5	−0.9	9	15
12	6ʹʹʹ-Sinapoylspinosin	814.7	0.6	9	19
13	6ʹʹʹ-*p*-Coumaloylspinosin	754.7	0.7	9	17
14	6ʹʹʹ-Feruloylspinosin	784.7	0.7	9	18
15	N-nornuciferine	281.3	3	1	3
16	Jujuboside A	1207.3	−1.6	14	26
17	Jujuboside B	1045.2	0.5	11	21
18	Ceanothic acid	486.7	7.6	3	5
19	Betulinic acid	456.7	8.2	2	3
20	Palmitic acid	256.42	6.4	1	2
21	Oleic acid	282.5	6.5	1	2

**TABLE 3:** Targets clustering analysis using MOCDE from PPI network.

Cluster	Score	Nodes number	Edges number	Targets
1	23	23	253	APP, CNR2, CNR1, HTR1E, HTR1F, ADORA1, SSTR2, SSTR5, HTR1B, ADRA2B, ADRA2C, ADRA2A, OPRM1, HTR1D, HTR5A, MTNR1B, DRD4, GABBR1, HTR1A, DRD3, DRD2, OPRD1, OPRK1
2	14.933	16	112	CHRM1, CHRM3, AGTR1, TACR1, ADRA1A, HTR3A, HTR2C, PTAFR, F2, ADRA1B, GRM5, HTR2B, ADRA1D, CCKBR, HTR2A, F2RL1
3	9.1	21	91	FGF2, HRH2, MMP9, HTR4, ADRB3, PPARG, ICAM1, ADRB2, ADRB1, TNF, AKT1, CASP3, DRD1, DRD5, HTR6, HTR7, VCAM1, SLC6A4, CCL2, PTGS2, VEGFA
4	5	5	10	GABRA1, GABRG2, GABRA2, GABRA5, GABRB2

#### GO and KEGG Pathway Enrichment Analysis

**FIGURE 4:**
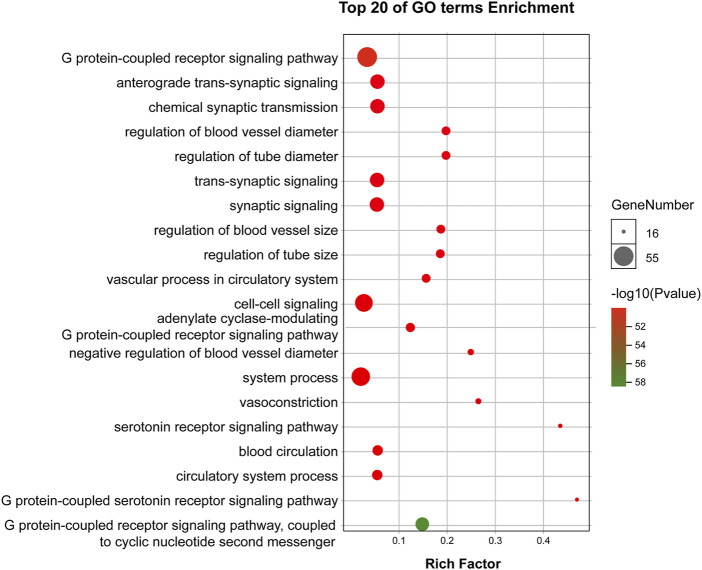
GO enrichment analysis of targets for ZSS in the treatment of insomnia.

**FIGURE 5:**
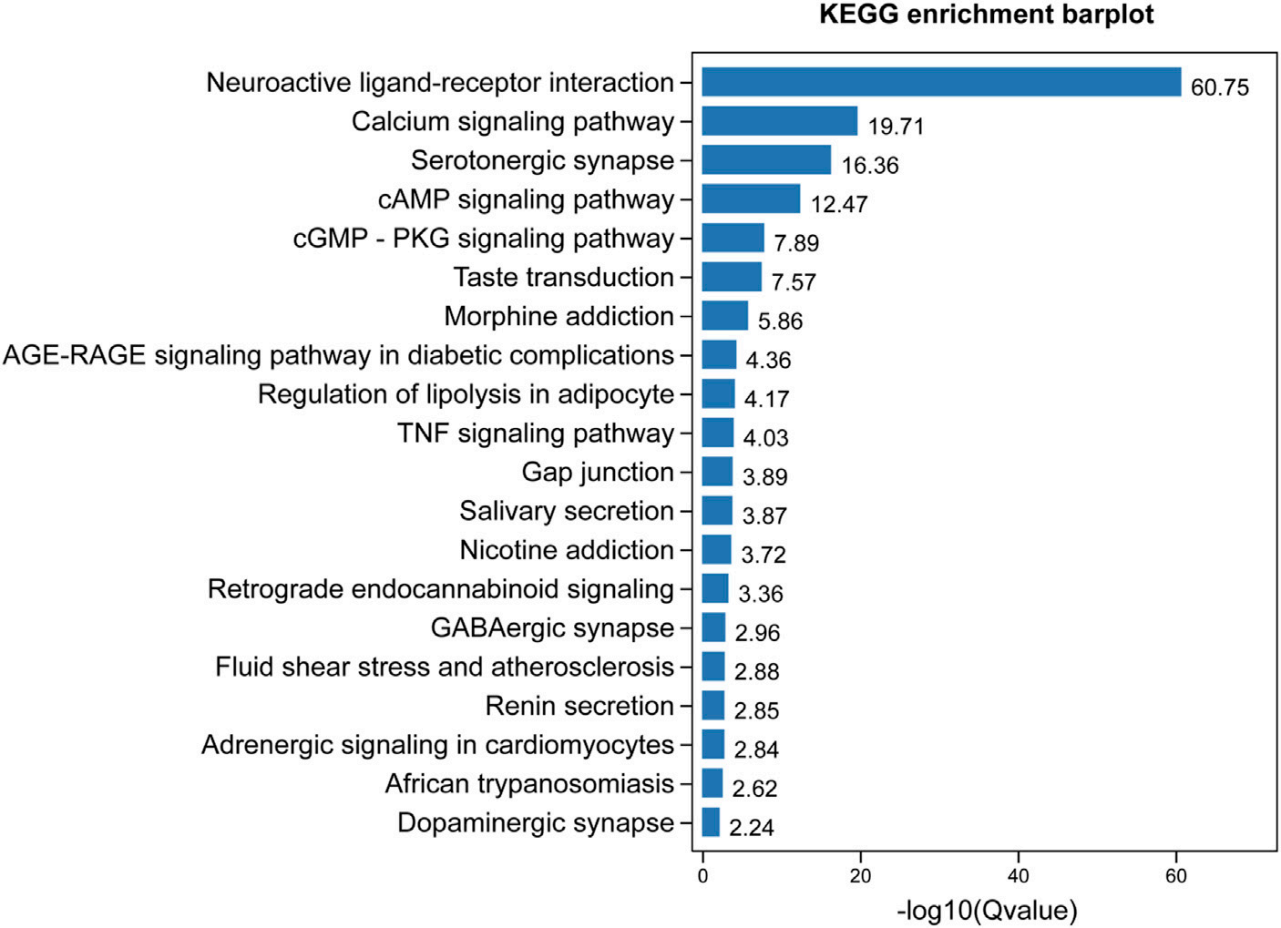
KEGG enrichment analysis of targets for ZSS in the treatment of insomnia.

GO enrichment analysis demonstrated that the core targets were associated with various neural-associated biological processes, including G protein-coupled receptor signaling pathways, anterograde *trans*-synaptic signaling, chemical synaptic transmission, *trans*-synaptic signaling, and synaptic signaling (Figure 4). Next, we applied KEGG enrichment analysis to identify the overall regulation of ZSS in the treatment of insomnia with regards to specific signaling pathways. The targets identified overlapped between neuroactive ligand-receptor interaction, serotonergic synapse, and GABAergic synapse (Figure 5).

**FIGURE 6:**
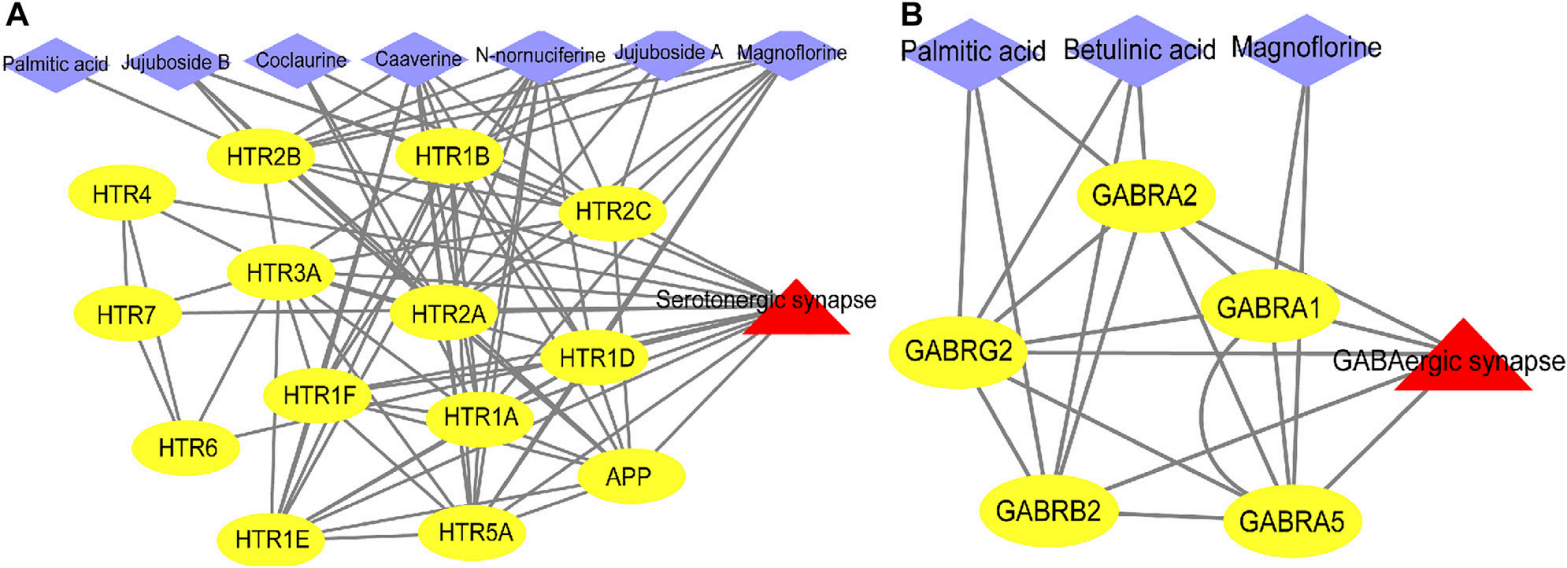
Ingredient-target-pathway (I-T-P) sub-networks of the serotonergic synapse pathway **(A)** and the GABAergic synapse pathway **(B)**.

Next, we used ingredient-target-pathway (I-T-P) sub-networks to construct serotonergic synapse and GABAergic synapse pathways and reveal the synergistic effects of ZSS to treat insomnia (Figures 6A,B). As shown in the two networks, palmitic acid, coclaurine, jujuboside A, N-nornuciferine, caaverine, magnoflorine, jujuboside B, and betulinic acid, all played roles in pathways associated with the serotonergic synapse and the GABAergic synapse.

Recent studies have shown that multiple subtypes of serotonin (5-HT) receptors In the central nervous system are mainly involved in the modulation of sleep (Cui et al., 2011). In addition, 5-HT_1A_ (HTR1A) and 5-HT_2A_ (HTR2A) have been identified as common targets for the treatment of insomnia, in both clinical and basic studies (Jiang et al., 2016; Ohno et al., 2018; Shao et al., 2020; Lv et al., 2021), as well as prevenient studies of ZSS for the treatment of insomnia (Shen et al., 2020). GABA_A_Rα1 (GABRA1) and GABA_A_Rγ2 (GABRG2) are two significant subunits of GABA receptors, and are known to play an important role in the first line treatment of insomnia (Zhong et al., 2020; Lv et al., 2021). Based on our preliminary results, we preferentially selected HTR1A, HTR2A, GABRA1, and GABRG2, as potential therapeutic targets of ZSS for further experimental validation.

### Experimental Validation

#### RT-qPCR Experiments

**FIGURE 7:**
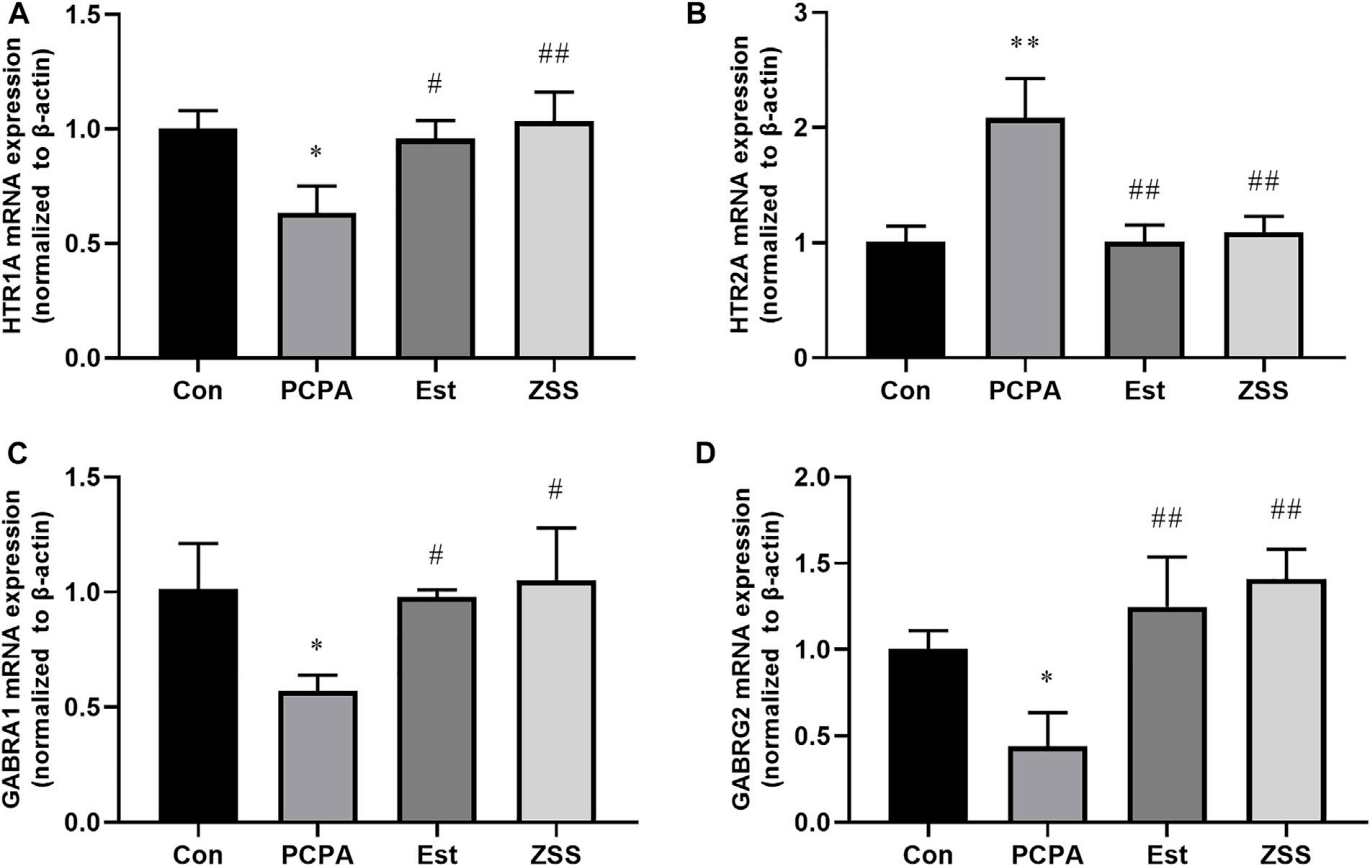
The effects of ZSS on the mRNA expression levels of *HTR1A* **(A)**, *HTR2A* **(B)**, *GABRA1* **(C)** and *GABRG2* **(D)** in the hypothalamus. ^*^ *p* < 0.05, ^**^ *p* < 0.01 compared with the control group, ^#^ *p* < 0.01, ^##^ *p* < 0.01 compared with the model group. Data are shown as mean ± SD values and n = 3.

Network pharmacology demonstrated that neuroactive ligand-receptor interaction (important-related targets: HTR1A, HTR2A, GABRA1 and GABRG2), serotonergic synapse (important-related targets: HTR1A and HTR2A), and GABAergic synapse (important-related targets: GABRA1 and GABRG2) signaling pathways were involved in the effects of ZSS on insomnia. We determined the expression levels of hypothalamic-related genes (Figures 7A–D). The administration of PCPA led to a significant reduction in the gene expression of *HTR1A*, *GABRA1*, and *GABRG2* (both *p* < 0.05), and an increase in the expression levels of *HTR2A* mRNA (*p* < 0.01). We found that ZSS treatment significantly elevated the gene expression levels of *HTR1A* (*p* < 0.01), *GABRA1* (*p* < 0.05) and *GABRG2* (*p* < 0.01) and decreased the expression levels of *HTR2A* mRNA (*p* < 0.01).

#### Immunohistochemistry

**FIGURE 8:**
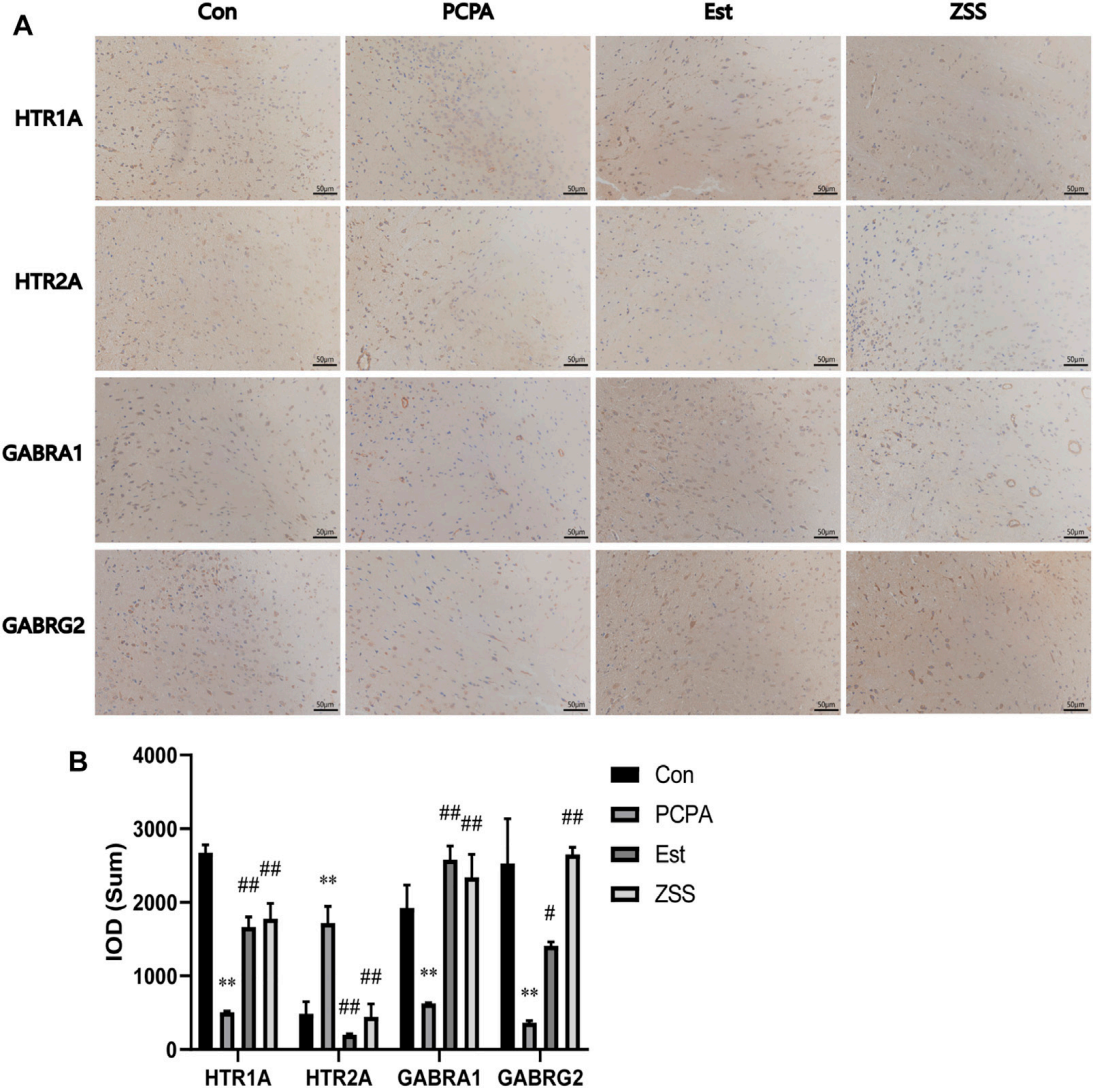
The effects of ZSS on the expression of HTR1A, HTR2A, GABRA1 and GABRG2 in the hypothalamus **(A)** and immunohistochemistry results (the sum of the IOD) **(B)**. ^**^ *p* < 0.01 compared with the control group, ^##^ *p* < 0.01 compared with the model group. Data are shown as mean ± SD values and n = 3.

We also used immunohistochemistry analysis to investigate the expression levels of related targets in the hypothalamus (Figures 8A,B). The injection of PCPA induced a significant reduction in the number of HTR1A-, GABRA1-, and GABRG2-positive cells (*p* < 0.01), and an increase in the number of HTR2A-positive cells in the hypothalamus (*p* < 0.01). Following ZSS treatment, the number of HTR1A-, GABRA1-, and GABRG2-positive cells increased significantly (*p* < 0.01); however, the number of HTR2A-positive cells decreased significantly (*p* < 0.01).

## Discussion

Previous research involving ZSS mostly focused on the pharmacodynamics of certain components, but failed to address the specific mechanisms underlying the pharmacodynamics of ZSS from an overall material basis. In this study, we employed network pharmacology and *in vivo* validation experiments to identify the specific mechanisms underlying the actions of ZSS in the treatment of insomnia. The identification of specific components of herbal materials identified by network pharmacology should be more accurate than when simply retrieved from databases of herbal ingredients (e.g., TCMSP, https://old.tcmsp-e.com/tcmsp.php; BATMAN-TCM, http://bionet.ncpsb.org.cn/batman-tcm/). The chemical composition of herbal materials may often change during the processes used for extraction and concentration. Research has shown that modern analytical techniques, such as GC-MS and UPLC-MS, should be used as supplementary tools to obtain more accurate results relating to chemical compositions (Ren et al., 2020; Tian et al., 2020; Oh et al., 2021). In the current study, UPLC-Q-Exactive-MS/MS identified a series of triterpenoid saponins and flavonoids from ZSS extract; some of these were spinosin derivatives, including 6‴-(4‴'-O-glc)-vanilloylspinosin, 6ʹʹʹ-pyridyloylspinosin, 6ʹʹʹ-*p*-hydroxylbenzoylspinosin, 6‴-O-(3S-1-N-β-D-glucopyranosyl-2-oxo-3-hydroxy-indole-3-acetyl) spinosyn, 6ʹʹʹ-sinapoylspinosin, 6ʹʹʹ-*p*-coumaloylspinosin, 6ʹʹʹ-feruloylspinosin, 6‴-(N-β-d-glucopyranosyl)-2‴',3‴'-dihydro-2‴'-oxo-3‴'-yl-acetate spinosin, 6-(-)-phaseolspinosin, 6ʹʹʹ-benzoylspinosin, and 6ʹʹ-O-(3-glc-indole-acetyl)-6ʹʹʹ-feruloylspinosin; these are rare in other plants. According to Lipinski’s rule of five, we selected compounds with good drug-likeness in ZSS for our network pharmacology research. In addition, we also included compounds with good activity but poor properties of drug-likeness.

Previous research identified that the serotonin (5-HT) system plays an active role in sleep disorders, depression, anxiety disorders, and other disorders of the central nervous system (Novati et al., 2008; Babson et al., 2017; Van Dalfsen and Markus, 2019). According to amino acid sequence, gene structure, second messengers, and pharmacological activity, the serotonin receptors can be divided into seven categories (HTR1-7) (Domínguez-Soto et al., 2017). The HTR1A and HTR2A subtypes are the most noteworthy receptors; this is because of their highly specific and important role in the brain. These receptor subtypes have been used to investigate the pathogenesis of nervous system diseases and research focused on anti-insomnia and anti-depressant drugs (Antypa et al., 2013; Dong et al., 2016). HTR1A receptor agonists, buspirone, and eptapirone, have all been shown to reduce REMS (rapid eyes movement sleep), increase REMS latency, and elevate the levels of 5-HT in brain (Wilson et al., 2005). This may be one of the reasons why HTR1A receptor agonists can ameliorate sleep symptoms. HTR1A receptors are mainly expressed on the axons of serotonergic neurons in the raphe nucleus, but are also expressed in the hypothalamus, thalamus, frontal cortex, amygdala and hippocampus (Polter and Li, 2010). The regulation of HTR1A receptors during sleep, emotion, self-cognition, and other physiological activities, involves the precise coordination of presynaptic and postsynaptic receptors (Albert et al., 2014). The results of both experimental and clinical studies have proven the effect of HTR2A receptor antagonists in the treatment of insomnia (Cohrs et al., 2005). HTR2A receptor antagonists (e.g., ketanserin, seganserinm, and ritanserin) can increase SWS (slow wave sleep) and NREMS (non-rapid eye movement sleep), reduce the number of awakenings, but has no effect on REMS. Therefore, HTR2A receptor antagonists are considered to be ideal drugs for insomnia (Fish et al., 2005; Yi et al., 2007; Monti, 2010). Similarly, our systems biology investigations showed that the serotonergic synapse signaling pathway plays a dominant role in the therapeutic effect of ZSS. Our RT-qPCR and immunohistochemistry experiments also demonstrated that ZSS extract had a significant regulatory effect on the expression of HTR1A and HTR2A.

GABA produces a neuroinhibitory effect by binding with its receptor GABA (A-C) (Bormann, 2000). Of the different forms of GABA receptor, the GABA_A_ receptor is the one that is most used in insomnia research. GABA acts on the GABA site of the GABA_A_ receptor; this increases the permeability of chloride ions in the membranes of nerve cells (Olsen and Sieghart, 2008). Chloride ions can then enter into cells along a concentration gradient; the negative potential in cell membranes increases further, from a polarized state to a hyperpolarized state (Dela Peña et al., 2015). Depolarization is very difficult and causes a reduction in excitability, thus resulting in sedation, hypnosis, and antianxiety (Succol et al., 2012). Of the GABA_A_ receptor subunits, GABRA1 and GABRG2 have been frequently reported to mediate insomnia (Tochitani and Kondo, 2013; Dixon et al., 2015). In this study, RT-qPCR and immunohistochemistry revealed that an extract of ZSS led to an increase in the expression of GABRA1 and GABRG2 receptors in the hypothalamus of rats suffering from insomnia. Systems biology analysis further indicated that the GABA_A_ receptor signaling pathway is a crucially important pathway involved in the therapeutic effect of ZSS.

The active ingredients of Chinese herbal materials (CHM) are the material basis for their pharmacological actions. In addition, these ingredients, with good biological activities and measurability, are considered as the quality markers (Q-markers) for CHM. Q-markers for CHM represent a new concept for the quality control of herbal medicines (Kang et al., 2019). As shown in our ingredients-target-pathway (I-T-P) sub-networks, two triterpenoid saponins (jujuboside A, jujuboside B), four alkaloids (coclaurine, N-nornuciferine, caaverine, magnoflorine), one triterpene acid (betulinic acid), and one fatty acid (palmitic acid), participated in regulating the serotonergic synapse and GABAergic synapse pathways. These components can therefore be regarded as the important material basis of ZSS for the treatment of insomnia, thus expanding our understanding of natural drugs for the treatment of insomnia. In addition to jujuboside A and spinosin as quality control indicators for Chinese Pharmacopoeia (Edition 2020), it is also important that we focus on alkaloids (coclaurine, N-nornuciferine, caaverine, and magnoflorine), and other core components (betulinic acid and palmitic acid), as candidate Q-markers.

## Conclusion

Our research indicated that the mechanism underlying the action of ZSS in the treatment of insomnia is mainly related to the modulation of 5-HT and GABAergic synapse pathways. We also demonstrated that the regulation of HTR1A, HTR2A, GABRA1, and GABRG2, plays significant roles in these pathways. Jujuboside A, jujuboside B, coclaurine, N-nornuciferine, caaverine, magnoflorine, betulinic acid, and palmitic acid, were all identified in ZSS and shown to contribute to the modulation of 5-HT and GABAergic synapse pathways. This research systematically investigated the role of multiple components in the effects of ZSS on insomnia and provided a new basis for the improvement of quality control standards. However, further in-depth preclinical studies are needed to validate the results obtained from the current analysis. These selected phytochemicals may be used as potential candidate drugs for the treatment of insomnia and deserve further research attention.

## Data Availability

The raw data supporting the conclusions of this article will be made available by the authors, without undue reservation.

## Abbreviations

5-HT, 5-hydroxytryptamine; ANOVA, Analysis of Variance; BATMAN-TCM, a bioinformatics analysis tool for molecular mechanism of traditional Chinese medicine; Con, control; DAB, 3,3′-diaminobenzidine; ESI-MS, electrospray ionization tandem mass spectrometry; Est, estazolam; GABA, Gamma-aminobutyric acid; GABRA1, Gamma-aminobutyric acid receptor subunit alpha-1; GABRG2, Gamma-aminobutyric acid receptor subunit gamma-2; GC-MS, gas chromatography-mass spectrometry; GO, gene ontology; HCD, high energy collision dissociation; HE, hematoxylin-eosin; HTR1A, 5-hydroxytryptamine receptor 1A; HTR2A, 5-hydroxytryptamine receptor 2A; IOD, integrated optical density; KEGG, Kyoto Encyclopedia of Genes and Genomes; LC-MS, liquid chromatography-mass spectrometry; MCODE, Molecular Complex Detection; mean ± SD, mean ± Standard deviation; PCPA, p-chlorophenylalanine; RT-qPCR, Real Time Quantitative PCR; TCMSP, traditional Chinese medicine systems pharmacology database and analysis platform; UHPLC-Q-Exactive-MS/MS, Ultra high performance liquid chromatography-quadrupole-exactive-mass spectrometry/mass spectrometry.
