# Supplementary material for: Mechanisms Underlying the Action of Ziziphi Spinosae Semen in the Treatment of Insomnia: A Study Involving Network Pharmacology and Experimental Validation
Source: Front Pharmacol. 2021 Dec 24;12:752211. doi: 10.3389/fphar.2021.752211 (PMC8740267; doi:10.3389/fphar.2021.752211)
Supplement: Supplementary file 1 [file DataSheet1.docx]

**Supplementary Table S1** Compound-related targets acquired in the ZSS extract.

| NO. | Targets | NO. | Targets | NO. | Targets | NO. | Targets | NO. | Targets | NO. | Targets | NO. | Targets | NO. | Targets | NO. | Targets |
| --- | --- | --- | --- | --- | --- | --- | --- | --- | --- | --- | --- | --- | --- | --- | --- | --- | --- |
| 1 | DRD2 | 22 | KCNH2 | 43 | TRPC3 | 64 | PTGES | 85 | TRPV1 | 106 | CCNA2 | 127 | CHRM4 | 148 | NMUR2 | 169 | LGALS8 |
| 2 | CHRNA4 | 23 | ADRA1B | 44 | JAK3 | 65 | CSF1R | 86 | TRPM8 | 107 | TBXA2R | 128 | CA7 | 149 | NQO2 | 170 | LGALS9 |
| 3 | DRD3 | 24 | TSPO | 45 | PTPN1 | 66 | MAPKAPK2 | 87 | PGR | 108 | ADRB3 | 129 | ABCB1 | 150 | NOX4 | 171 | SLC29A1 |
| 4 | DRD1 | 25 | SLC6A4 | 46 | SIGMAR1 | 67 | KDR | 88 | MMP3 | 109 | F3 | 130 | SLC18A2 | 151 | CA4 | 172 | ADORA1 |
| 5 | HTR1A | 26 | ADRA2A | 47 | PIM1 | 68 | CDK1 | 89 | MMP2 | 110 | KCNN1 | 131 | JAK1 | 152 | PTGS2 | 173 | TYR |
| 6 | HTR6 | 27 | ADRA2C | 48 | DYRK1A | 69 | IRAK4 | 90 | ADORA2A | 111 | KCNN3 | 132 | JAK2 | 153 | RPS6KA3 | 174 | IMPDH1 |
| 7 | HTR7 | 28 | ADRA2B | 49 | STAT3 | 70 | ALOX15 | 91 | MAPK1 | 112 | KCNN2 | 133 | TYK2 | 154 | PDE5A | 175 | IMPDH2 |
| 8 | PTPRCAP | 29 | HTR1B | 50 | CLK1 | 71 | ALOX12 | 92 | PDE10A | 113 | MAOA | 134 | KDM1A | 155 | CD38 | 176 | HASPIN |
| 9 | HTR2B | 30 | HRH2 | 51 | DYRK2 | 72 | PIK3CB | 93 | CXCR3 | 114 | OPRK1 | 135 | CA1 | 156 | IL2 | 177 | CDK9 |
| 10 | ADRA1D | 31 | PPARG | 52 | PRKCG | 73 | PIK3CG | 94 | MMP9 | 115 | RBBP9 | 136 | JUN | 157 | ALOX5 | 178 | CYP1A2 |
| 11 | HTR2A | 32 | ADRB2 | 53 | GABRA1 | 74 | PIK3CA | 95 | PDGFRA | 116 | HCRTR2 | 137 | CA2 | 158 | HSP90AA1 | 179 | CHRNB1 |
| 12 | TH | 33 | CDK5R1 | 54 | EPHX2 | 75 | HSD17B3 | 96 | AURKB | 117 | HCRTR1 | 138 | GRIK2 | 159 | MMP13 | 180 | HTR1F |
| 13 | DRD4 | 34 | GSK3B | 55 | VCAM1 | 76 | PDGFRB | 97 | ROCK2 | 118 | ACHE | 139 | CA9 | 160 | MMP1 | 181 | CHRNB3 |
| 14 | HTR5A | 35 | CDK2 | 56 | GABRA5 | 77 | FLT4 | 98 | FGFR1 | 119 | DPP4 | 140 | HDAC1 | 161 | MMP7 | 182 | NR4A1 |
| 15 | ADRA1A | 36 | NR3C2 | 57 | MAP2K1 | 78 | SRC | 99 | AKT1 | 120 | OPRD1 | 141 | MTNR1B | 162 | MMP12 | 183 | SCN4A |
| 16 | HTR1D | 37 | EGFR | 58 | ADAMTS5 | 79 | ICAM1 | 100 | CHRNB2 | 121 | DHCR7 | 142 | ROCK1 | 163 | MMP8 | 184 | HTR1E |
| 17 | ADRB1 | 38 | HPGD | 59 | RPS6KB1 | 80 | SELE | 101 | CDC7 | 122 | SLC6A2 | 143 | CHRNA2 | 164 | SLC5A4 | 185 | LTA4H |
| 18 | SLC6A3 | 39 | AR | 60 | KAT2B | 81 | PPP5C | 102 | CCNB1 | 123 | SLC47A1 | 144 | AKR1B1 | 165 | SLC5A1 | 186 | HTR4 |
| 19 | OPRM1 | 40 | CYP19A1 | 61 | SIRT2 | 82 | CCNB3 | 103 | CCNB2 | 124 | CHRNB4 | 145 | CA12 | 166 | LGALS3 | 187 | AOC3 |
| 20 | DRD5 | 41 | MGLL | 62 | FLT3 | 83 | DBF4 | 104 | CDK5 | 125 | CHRNA3 | 146 | TNF | 167 | CA14 | 188 | HTR3A |
| 21 | HTR2C | 42 | TRPC6 | 63 | CLK3 | 84 | AURKA | 105 | CCNA1 | 126 | BCHE | 147 | XDH | 168 | LGALS4 | 189 | FAP |

**Supplementary Table S1** Compound-related targets acquired in the ZSS extract.

| NO. | Targets | NO. | Targets | NO. | Targets | NO. | Targets | NO. | Targets | NO. | Targets | NO. | Targets | NO. | Targets | NO. | Targets | NO. | Targets |
| --- | --- | --- | --- | --- | --- | --- | --- | --- | --- | --- | --- | --- | --- | --- | --- | --- | --- | --- | --- |
| 190 | DPP9 | 211 | CHRM2 | 232 | PPP2R5A | 253 | PRKCQ | 274 | VDR | 295 | CASP3 | 316 | CNR1 | 337 | TACR1 | 358 | FABP1 | 379 | ALOX5AP |
| 191 | IKBKB | 212 | CHRM1 | 233 | VEGFA | 254 | RAP1A | 275 | TLR9 | 296 | CASP1 | 317 | CNR2 | 338 | HDAC8 | 359 | HCAR2 | 380 | GABBR1 |
| 192 | MAPK8 | 213 | CHRM3 | 234 | FGF1 | 255 | PDE4D | 276 | SF3B3 | 297 | IGF1R | 318 | HLA-DRB1 | 339 | SLC5A2 | 360 | MME | 381 | PTPN11 |
| 193 | CTSC | 214 | CYP2D6 | 235 | FGF2 | 256 | ABCG2 | 277 | CDC25A | 298 | GRB2 | 319 | P2RY12 | 340 | CMA1 | 361 | ECE1 | 382 | TERT |
| 194 | PLA2G2A | 215 | CHRNA6 | 236 | HPSE | 257 | EDNRA | 278 | CDC25B | 299 | HLA-A | 320 | APP | 341 | ITGA4 | 362 | ESR2 | 383 | NPC1L1 |
| 195 | CCL2 | 216 | CHRNA1 | 237 | BCL2L1 | 258 | GLI1 | 279 | GAA | 300 | LCK | 321 | CCKBR | 342 | ITGB6 | 363 | ADAMTS4 | 384 | CYP17A1 |
| 196 | MYLK | 217 | CHRNG | 238 | RORC | 259 | TYMS | 280 | MLNR | 301 | PTPN22 | 322 | XIAP | 343 | ITGA5 | 364 | SERPINA6 | 385 | GABRA2 |
| 197 | PKN2 | 218 | CHRND | 239 | ATP1A1 | 260 | DHFR | 281 | FNTA | 302 | RRM1 | 323 | TOP1 | 344 | ITGB3 | 365 | SHBG | 386 | FABP2 |
| 198 | PKN1 | 219 | CHRNA7 | 240 | PSEN2 | 261 | METAP2 | 282 | PGGT1B | 303 | ITGAV | 324 | AGTR1 | 345 | POLB | 366 | G6PD | 387 | PTPN6 |
| 199 | PRKCE | 220 | NOS2 | 241 | HSD11B2 | 262 | CXCR1 | 283 | SSTR5 | 304 | HLA-DRB3 | 325 | PLG | 346 | AKR1B10 | 367 | CYP51A1 | 388 | GABRB2 |
| 200 | RPS6KA5 | 221 | LRRK2 | 242 | HSD11B1 | 263 | PTPA | 284 | SSTR2 | 305 | LTB4R | 326 | F10 | 347 | SAE1 | 368 | GPBAR1 | 389 | GABRG2 |
| 201 | PHLPP2 | 222 | PRMT6 | 243 | PRKCA | 264 | AMY2A | 285 | SSTR4 | 306 | CTSB | 327 | F7 | 348 | NR1H4 | 369 | AMPD2 | 390 | FABP4 |
| 202 | ADCY5 | 223 | PRMT8 | 244 | PLA2G1B | 265 | AMY1A | 286 | SSTR1 | 307 | ITGA2B | 328 | ITGB7 | 349 | TOP2A | 370 | CTSA | 391 | FABP3 |
| 203 | IDO1 | 224 | AXL | 245 | GLRA1 | 266 | F2RL1 | 287 | SSTR3 | 308 | BACE1 | 329 | ITGB5 | 350 | CDC25C | 371 | SCD | 392 | FABP5 |
| 204 | PRKX | 225 | TYRO3 | 246 | GLRA2 | 267 | IARS | 288 | PSENEN | 309 | ITGB1 | 330 | TACR3 | 351 | CES2 | 372 | CHEK1 | 393 | PPARD |
| 205 | QDPR | 226 | MERTK | 247 | REN | 268 | GCGR | 289 | NCSTN | 310 | TACR2 | 331 | FANCF | 352 | PREP | 373 | WEE1 | 394 | FFAR1 |
| 206 | KISS1R | 227 | PRMT1 | 248 | F2 | 269 | CCR6 | 290 | APH1A | 311 | PTPN2 | 332 | LNPEP | 353 | UGT2B7 | 374 | PTGER4 | 395 | SLC22A6 |
| 207 | PIM2 | 228 | PTAFR | 249 | TTL | 270 | SQLE | 291 | PSEN1 | 312 | PTPRA | 333 | PPARA | 354 | NR1H3 | 375 | PLA2G4A | 396 | PHF8 |
| 208 | FDFT1 | 229 | PPM1B | 250 | PRKCD | 271 | PPM1A | 292 | APH1B | 313 | CAPN1 | 334 | MDM2 | 355 | PTGER2 | 376 | UBA2 | 397 | KDM2A |
| 209 | HRH4 | 230 | PPP1CC | 251 | PRKCB | 272 | KCNA3 | 293 | FNTB | 314 | MAGI3 | 335 | HDAC6 | 356 | ACE | 377 | PTPRF | 398 | KDM5C |
| 210 | TNKS2 | 231 | PPP2CA | 252 | PRKCH | 273 | ATP6AP1 | 294 | CASP9 | 315 | IDE | 336 | FKBP1A | 357 | CD81 | 378 | ACP1 | 399 | HAO1 |

**Supplementary Table S1** Compound-related targets acquired in the ZSS extract.

| NO. | Targets | NO. | Targets |
| --- | --- | --- | --- |
| 400 | PTGFR | 421 | GRM5 |
| 401 | GSTK1 | 422 | HNF4A |
| 402 | NR0B2 | 423 | PTGS1 |
| 403 | CDC45 | 424 | NR3C1 |
| 404 | PTPRC | 425 | PTGIR |
| 405 | RXRA | 426 | ADORA3 |
| 406 | CYP26A1 | 427 | MAPK3 |
| 407 | CYP26B1 | 428 | PTGER1 |
| 408 | RARG | 429 | NR1I3 |
| 409 | RARB | 430 | CYP2C19 |
| 410 | RARA | 431 | SRD5A2 |
| 411 | FAAH | 432 | PDE4A |
| 412 | FFAR4 | 433 | PDE4B |
| 413 | CACNA2D1 |  |  |
| 414 | RXRG |  |  |
| 415 | RXRB |  |  |
| 416 | SLC16A1 |  |  |
| 417 | ABCC1 |  |  |
| 418 | HMGCR |  |  |
| 419 | RORB |  |  |
| 420 | RORA |  |  |

**Supplementary Table S2** Insomnia-associated targets gathered from the GeneCards database.

| NO. | Targets | NO. | Targets | NO. | Targets | NO. | Targets | NO. | Targets | NO. | Targets | NO. | Targets | NO. | Targets |
| --- | --- | --- | --- | --- | --- | --- | --- | --- | --- | --- | --- | --- | --- | --- | --- |
| 1 | ADORA2A | 22 | PDE4B | 43 | OPRK1 | 64 | FADS1 | 85 | ALAD | 106 | GRIN1 | 127 | OTC | 148 | HTR2B |
| 2 | ADORA1 | 23 | XDH | 44 | MUC2 | 65 | ADH1C | 86 | PLAT | 107 | NR3C2 | 128 | CYP19A1 | 149 | HTR1B |
| 3 | CHRM1 | 24 | DNMT1 | 45 | IL5 | 66 | PTGS1 | 87 | ABAT | 108 | CCKAR | 129 | SRR | 150 | NCF1 |
| 4 | KCNMA1 | 25 | NOS1 | 46 | PRKCA | 67 | COX6B1 | 88 | SCN1A | 109 | GABRB1 | 130 | CHRNA4 | 151 | HTR2C |
| 5 | ABCC2 | 26 | ABCC8 | 47 | OPRM1 | 68 | CES1 | 89 | CYP17A1 | 110 | SLC52A2 | 131 | CHRNA7 | 152 | DRD2 |
| 6 | FBP1 | 27 | ALK | 48 | CNR1 | 69 | PTGS2 | 90 | CACNA1A | 111 | ITGAL | 132 | ACHE | 153 | SLC6A4 |
| 7 | AKT1 | 28 | ABCB11 | 49 | ACE | 70 | ADH1B | 91 | CACNA2D2 | 112 | OAZ3 | 133 | KCNH2 | 154 | HTR7 |
| 8 | GSR | 29 | ABCB1 | 50 | PPARD | 71 | HMGCR | 92 | GRIN2A | 113 | LIAS | 134 | BCHE | 155 | HRH2 |
| 9 | ADCY1 | 30 | ADRB2 | 51 | MME | 72 | AKR1C3 | 93 | PPARG | 114 | P3H3 | 135 | CHRFAM7A | 156 | HTR1F |
| 10 | MMAB | 31 | MTNR1A | 52 | CREBBP | 73 | SLC25A20 | 94 | ALDH5A1 | 115 | RARRES1 | 136 | CHRNB4 | 157 | HTR1A |
| 11 | NT5C2 | 32 | OPRD1 | 53 | SLC6A3 | 74 | MPO | 95 | SCN2A | 116 | SLC25A15 | 137 | KCNA5 | 158 | PGRMC1 |
| 12 | AMHR2 | 33 | TLR4 | 54 | PNKD | 75 | AGTR1 | 96 | NR3C1 | 117 | SHBG | 138 | NPPA | 159 | DRD3 |
| 13 | MTRR | 34 | CYSLTR1 | 55 | NMT1 | 76 | SLC22A5 | 97 | PLG | 118 | SLC13A3 | 139 | RYR2 | 160 | HTR1E |
| 14 | ADK | 35 | TUBB | 56 | REC8 | 77 | VDR | 98 | HDAC9 | 119 | NOS2 | 140 | CASQ2 | 161 | DRD1 |
| 15 | MAOB | 36 | ESR1 | 57 | TSR1 | 78 | CYP27B1 | 99 | CACNA1B | 120 | SDHB | 141 | GABRA2 | 162 | SLC6A2 |
| 16 | MTR | 37 | CNR2 | 58 | WAC | 79 | ACO1 | 100 | GRIN2C | 121 | CACNA2D3 | 142 | CHRM3 | 163 | CNTFR |
| 17 | MAOA | 38 | TNF | 59 | RELA | 80 | CRP | 101 | GRIN2B | 122 | CTPS1 | 143 | GRIA2 | 164 | SLC5A7 |
| 18 | AIFM1 | 39 | MTNR1B | 60 | COX5A | 81 | NR0B1 | 102 | LARS2 | 123 | CACNA1C | 144 | GABRA1 | 165 | TAC1 |
| 19 | CREB1 | 40 | ADRB1 | 61 | AR | 82 | IREB2 | 103 | SCN8A | 124 | CACNA1D | 145 | HTR4 | 166 | CDH2 |
| 20 | PYGL | 41 | RNASE3 | 62 | FECH | 83 | BAX | 104 | SCN1B | 125 | SLC25A2 | 146 | ADRA1A | 167 | CCL5 |
| 21 | ASS1 | 42 | RARB | 63 | FADS2 | 84 | INS | 105 | GABBR1 | 126 | CLCN2 | 147 | HTR2A | 168 | NTRK1 |

**Supplementary Table S2** Insomnia-associated targets gathered from the GeneCards database.

| NO. | Targets | NO. | Targets | NO. | Targets | NO. | Targets | NO. | Targets | NO. | Targets | NO. | Targets | NO. | Targets |
| --- | --- | --- | --- | --- | --- | --- | --- | --- | --- | --- | --- | --- | --- | --- | --- |
| 169 | AGT | 190 | KISS1 | 211 | HCN1 | 232 | GABRG1 | 253 | HTR6 | 274 | IFNG | 295 | SOD1 | 316 | GLO1 |
| 170 | RUNX1 | 191 | F2 | 212 | HMGA2 | 233 | GABRP | 254 | ELANE | 275 | PAM | 296 | SOD2 | 317 | CLU |
| 171 | CAT | 192 | CCKBR | 213 | TNFSF11 | 234 | GABRA4 | 255 | FGFR2 | 276 | MTOR | 297 | FURIN | 318 | TLN2 |
| 172 | NLGN4X | 193 | LHCGR | 214 | ASMT | 235 | THRA | 256 | PDE5A | 277 | FGF2 | 298 | UGT1A1 | 319 | WARS2 |
| 173 | NPS | 194 | SSTR5 | 215 | PRKCG | 236 | DDC | 257 | HNMT | 278 | VCAM1 | 299 | SLCO2B1 | 320 | TTR |
| 174 | TNNI3K | 195 | SSTR2 | 216 | PRKCD | 237 | HRH1 | 258 | S100A12 | 279 | SLC29A1 | 300 | OCA2 | 321 | PCDH1 |
| 175 | ADRA1D | 196 | TACR1 | 217 | ADRA2A | 238 | SLC18A2 | 259 | TUBB2B | 280 | HTR3B | 301 | SERPINE1 | 322 | TPK1 |
| 176 | AIF1 | 197 | OXTR | 218 | CFTR | 239 | GABRQ | 260 | VEGFA | 281 | GRIA3 | 302 | RAB7A | 323 | ADA |
| 177 | ANK3 | 198 | ALB | 219 | DHFR | 240 | DRD4 | 261 | TUBB2A | 282 | SLC29A2 | 303 | CHGA | 324 | SERPINC1 |
| 178 | COLQ | 199 | AVPR1B | 220 | ADRA2C | 241 | DBH | 262 | TUBA1B | 283 | GLRA1 | 304 | REST | 325 | GAD1 |
| 179 | ASCL1 | 200 | GNRHR | 221 | PDE10A | 242 | THRB | 263 | CHAT | 284 | PSG3 | 305 | HRH3 | 326 | GCH1 |
| 180 | PHOX2B | 201 | GRIA4 | 222 | PDE2A | 243 | KCNJ5 | 264 | S100B | 285 | MGRN1 | 306 | SELE | 327 | RIN2 |
| 181 | ADRA1B | 202 | TUBB3 | 223 | ADRA2B | 244 | GABRA5 | 265 | GNAO1 | 286 | TMEM132E | 307 | HIF1A | 328 | PEG10 |
| 182 | IL10 | 203 | CYP3A5 | 224 | GABRD | 245 | DRD5 | 266 | HTR5A | 287 | WDR45 | 308 | NPPB | 329 | TRAPPC9 |
| 183 | KCNIP1 | 204 | GRIA1 | 225 | GABRG3 | 246 | GABRA6 | 267 | NTRK3 | 288 | ATF4 | 309 | CFH | 330 | WARS1 |
| 184 | AGRN | 205 | ESR2 | 226 | TH | 247 | KCNJ1 | 268 | MAP2 | 289 | TSN | 310 | HPX | 331 | ALX3 |
| 185 | TSPO | 206 | CYP3A4 | 227 | TPO | 248 | GABRG2 | 269 | ARRB2 | 290 | AKR1A1 | 311 | CASP3 | 332 | PART1 |
| 186 | CALCA | 207 | CYP51A1 | 228 | CYP11A1 | 249 | ADRB3 | 270 | YWHAZ | 291 | HSP90B1 | 312 | KLK3 | 333 | FRAXA |
| 187 | HDAC4 | 208 | GABRB2 | 229 | GABRE | 250 | HTR1D | 271 | CCL2 | 292 | MAPT | 313 | RANBP3 | 334 | IL13 |
| 188 | GRIK1 | 209 | GABRB3 | 230 | GABRA3 | 251 | CALY | 272 | P4HTM | 293 | TPH1 | 314 | COMT | 335 | GH1 |
| 189 | NEFH | 210 | SLCO1B1 | 231 | PIK3R1 | 252 | HTR3A | 273 | MMP9 | 294 | NOS3 | 315 | APOE | 336 | PRL |

**Supplementary Table S2** Insomnia-associated targets gathered from the GeneCards database.

| NO. | Targets | NO. | Targets | NO. | Targets | NO. | Targets | NO. | Targets | NO. | Targets | NO. | Targets | NO. | Targets |
| --- | --- | --- | --- | --- | --- | --- | --- | --- | --- | --- | --- | --- | --- | --- | --- |
| 337 | LOC110806262 | 358 | VWF | 379 | IL18 | 400 | C5AR1 | 421 | CYP2A6 | 442 | PARP1 | 463 | EYA1 | 484 | PYY |
| 338 | POMC | 359 | ITCH | 380 | UGT2B15 | 401 | FCER1A | 422 | F3 | 443 | APOA1 | 464 | HNRNPK | 485 | GNRH1 |
| 339 | PPARGC1A | 360 | ACADVL | 381 | PPIG | 402 | CSF1 | 423 | NPY | 444 | IL1RN | 465 | GRM3 | 486 | MLXIPL |
| 340 | LOC110973015 | 361 | MB | 382 | UGT1A3 | 403 | SMN2 | 424 | IL2 | 445 | CAMK2G | 466 | RFC1 | 487 | GRP |
| 341 | KCNK3 | 362 | GPT | 383 | UGT1A8 | 404 | IL7 | 425 | CRH | 446 | GK | 467 | HCN2 | 488 | NMUR1 |
| 342 | GUSB | 363 | GSTP1 | 384 | UGT1A | 405 | SST | 426 | CGA | 447 | BCL2L1 | 468 | NPY1R | 489 | TFF3 |
| 343 | HBB | 364 | SLC17A5 | 385 | ENO2 | 406 | ICAM3 | 427 | NAT2 | 448 | MTHFR | 469 | IGFBP3 | 490 | DIO2 |
| 344 | KCNQ2 | 365 | CXCL8 | 386 | REN | 407 | CCL17 | 428 | F2RL2 | 449 | GRM5 | 470 | AASS | 491 | GAST |
| 345 | KCNQ3 | 366 | IL4 | 387 | CYP1A2 | 408 | IGES | 429 | HCRT | 450 | UBE3A | 471 | GRM2 | 492 | SCGB1A1 |
| 346 | STXBP1 | 367 | OXA1L | 388 | TP53 | 409 | BDNF | 430 | GPR55 | 451 | LBR | 472 | ELN | 493 | RSU1 |
| 347 | KCNA2 | 368 | LACTB | 389 | IL1B | 410 | RELN | 431 | OXT | 452 | LEP | 473 | KCNC3 | 494 | OPN4 |
| 348 | KCNA1 | 369 | CYP2D6 | 390 | BECN1 | 411 | CCK | 432 | SCT | 453 | FOLH1 | 474 | IL3 | 495 | EPRS1 |
| 349 | CDKL5 | 370 | IGHE | 391 | TPT1 | 412 | CYP2C19 | 433 | LOC110011216 | 454 | DHCR7 | 475 | THBD | 496 | SAMD9L |
| 350 | KCNA4 | 371 | PIK3C2A | 392 | ABCC3 | 413 | CYP2C9 | 434 | HMOX1 | 455 | SLC25A13 | 476 | VIP | 497 | DENND1B |
| 351 | KCNB2 | 372 | NGF | 393 | HSPG2 | 414 | FAAH | 435 | PSEN1 | 456 | F5 | 477 | GALC | 498 | NPY4R |
| 352 | PCDH19 | 373 | ICAM1 | 394 | P2RX7 | 415 | LCN2 | 436 | APP | 457 | AVP | 478 | FMO1 | 499 | CGB5 |
| 353 | FASTK | 374 | NISCH | 395 | CXCL10 | 416 | GABRR1 | 437 | PLAU | 458 | FCER2 | 479 | HFE | 500 | INTS11 |
| 354 | PRRT2 | 375 | NTS | 396 | SLC22A2 | 417 | GABRR2 | 438 | ALDH2 | 459 | A2M | 480 | IL18R1 | 501 | OPRPN |
| 355 | PCDH10 | 376 | CSF2 | 397 | KITLG | 418 | TBC1D24 | 439 | IGF1 | 460 | ADCY10 | 481 | PANK2 | 502 | RNY5 |
| 356 | PTGDS | 377 | F2RL1 | 398 | IL1A | 419 | DSPP | 440 | CAMK2A | 461 | CXCL12 | 482 | UGT1A9 | 503 | LOC108449888 |
| 357 | IL6 | 378 | SELP | 399 | ORAI1 | 420 | GABRR3 | 441 | PSEN2 | 462 | CYP2E1 | 483 | PTAFR | 504 | LOC108449897 |

**Supplementary** **Table S3** Information of potential targets of ZSS extract in the treatment of insomnia.

| NO. | Targets | NO. | Targets | NO. | Targets | NO. | Targets | NO. | Targets | NO. | Targets |
| --- | --- | --- | --- | --- | --- | --- | --- | --- | --- | --- | --- |
| 1 | DRD2 | 22 | ADRA1B | 43 | MMP9 | 64 | ADORA1 | 85 | PRKCD | 106 | CYP17A1 |
| 2 | CHRNA4 | 23 | TSPO | 44 | AKT1 | 65 | CYP1A2 | 86 | DHFR | 107 | GABRA2 |
| 3 | DRD3 | 24 | SLC6A4 | 45 | ADRB3 | 66 | HTR1F | 87 | F2RL1 | 108 | PPARD |
| 4 | DRD1 | 25 | ADRA2A | 46 | F3 | 67 | HTR1E | 88 | VDR | 109 | RARB |
| 5 | HTR1A | 26 | ADRA2C | 47 | MAOA | 68 | HTR4 | 89 | SSTR5 | 110 | FAAH |
| 6 | HTR6 | 27 | ADRA2B | 48 | OPRK1 | 69 | HTR3A | 90 | SSTR2 | 111 | HMGCR |
| 7 | HTR7 | 28 | HTR1B | 49 | ACHE | 70 | CCL2 | 91 | PSEN1 | 112 | GRM5 |
| 8 | HTR2B | 29 | HRH2 | 50 | OPRD1 | 71 | CHRM1 | 92 | CASP3 | 113 | GABRB2 |
| 9 | ADRA1D | 30 | PPARG | 51 | DHCR7 | 72 | CHRM3 | 93 | CNR1 | 114 | GABRG2 |
| 10 | HTR2A | 31 | ADRB2 | 52 | SLC6A2 | 73 | CYP2D6 | 94 | CNR2 | 115 | PTGS1 |
| 11 | TH | 32 | NR3C2 | 53 | CHRNB4 | 74 | CHRNA7 | 95 | APP | 116 | NR3C1 |
| 12 | DRD4 | 33 | AR | 54 | BCHE | 75 | NOS2 | 96 | CCKBR | 117 | CYP2C19 |
| 13 | HTR5A | 34 | CYP19A1 | 55 | ABCB1 | 76 | PTAFR | 97 | AGTR1 | 118 | PDE4B |
| 14 | ADRA1A | 35 | PRKCG | 56 | SLC18A2 | 77 | VEGFA | 98 | PLG |  |  |
| 15 | HTR1D | 36 | GABRA1 | 57 | MTNR1B | 78 | FGF2 | 99 | TACR1 |  |  |
| 16 | ADRB1 | 37 | VCAM1 | 58 | TNF | 79 | BCL2L1 | 100 | ACE |  |  |
| 17 | SLC6A3 | 38 | GABRA5 | 59 | XDH | 80 | PSEN2 | 101 | MME |  |  |
| 18 | OPRM1 | 39 | ICAM1 | 60 | PTGS2 | 81 | PRKCA | 102 | ESR2 |  |  |
| 19 | DRD5 | 40 | SELE | 61 | PDE5A | 82 | GLRA1 | 103 | SHBG |  |  |
| 20 | HTR2C | 41 | ADORA2A | 62 | IL2 | 83 | REN | 104 | CYP51A1 |  |  |
| 21 | KCNH2 | 42 | PDE10A | 63 | SLC29A1 | 84 | F2 | 105 | GABBR1 |  |  |
